# Checkpoint inhibitors in metastatic gastric and GEJ cancer: a multi-institutional retrospective analysis of real-world data in a Western cohort

**DOI:** 10.1186/s12885-021-09115-6

**Published:** 2022-01-10

**Authors:** Verena Schlintl, Florian Huemer, Gabriel Rinnerthaler, Thomas Melchardt, Thomas Winder, Patrick Reimann, Jakob Riedl, Arno Amann, Wolfgang Eisterer, Franz Romeder, Gudrun Piringer, Aysegül Ilhan-Mutlu, Ewald Wöll, Richard Greil, Lukas Weiss

**Affiliations:** 1 Department of Internal Medicine III with Haematology, Medical Oncology, Haemostaseology, Infectiology and Rheumatology, Oncologic Center, Salzburg Cancer Research Institute - Laboratory for Immunological and Molecular Cancer Research (SCRI-LIMCR), Center for Clinical Cancer and Immunology Trials (CCCIT), Paracelsus Medical University, Salzburg, Austria; 2 Department of Internal Medicine II, Academic Teaching Hospital Feldkirch, Feldkirch, Austria; 3 Division of Clinical Oncology, Department of Internal Medicine, Medical University of Graz, Graz, Austria; 4 Department of Internal Medicine V, Medical University Innsbruck, Innsbruck, Austria; 5 Department of Internal Medicine and Oncology, Klagenfurt Hospital, Klagenfurt, Austria; 6 Internal Medicine I: Department of Medical Oncology and Haematology, Ordensklinikum Linz Barmherzige Schwestern, Linz, Austria; 7 Department of Internal Medicine IV, Wels-Grieskirchen Hospital, Wels, Austria and Johannes Kepler University Linz, Linz, Austria; 8 Department of Medicine I, Comprehensive Cancer Center Vienna, Gastroesophageal Tumor Unit, Medical University of Vienna, Vienna, Austria; 9 Department of Internal Medicine, St. Vinzenz Hospital, Zams, Austria

**Keywords:** Gastric cancer, Nivolumab, Pembrolizumab, Immunotherapy

## Abstract

**Background:**

Safety and efficacy of immune checkpoint inhibitors in advanced gastric or gastroesophageal junction (GEJ) cancer could be demonstrated in predominantly Asian cohorts, whereas data in Western patients outside of clinical trials are vastly missing.

**Methods:**

In this multi-institutional retrospective analysis conducted at nine oncologic centers in Austria, we tried to assess feasibility of checkpoint inhibitors in advanced gastric/GEJ cancer in a real-world Western cohort.

**Results:**

In total, data from 50 patients with metastatic gastric/GEJ cancer who received nivolumab or pembrolizumab in a palliative setting between November 2015 and April 2020 have been evaluated. The median number of previous palliative therapy lines was two. The median progression-free survival (PFS) and overall survival (OS) were 2.1 (95% CI: 1.4**–**2.8) and 6.3 (95% CI: 3.3**–**9.3) months, respectively. There was no statistically significant difference in median OS according to microsatellite or PD-L1 status. However, a trend towards prolonged PFS and OS for the microsatellite instability high subgroup could be observed. Patients with an ECOG Performance Status (PS) ≥ 2 displayed a significantly worse outcome than those with an ECOG PS ≤ 1 (***p*** = .03). Only one patient discontinued immunotherapy due to treatment-related toxicity.

**Conclusions:**

Our results support feasibility of nivolumab and pembrolizumab in pre-treated patients with metastatic gastric and GEJ cancer in a Western real-world cohort. Further phase II/III studies are needed to confirm clinical efficacy.

## Background

Gastric and gastroesophageal junction (GEJ) adenocarcinomas show a cancer-specific mortality of 70% and thereby represent a substantial cause of cancer-related death worldwide [1]. Despite a decrease in annual incidence of new cases in Western patients during the last decade [2], diagnosis is still often established in advanced or metastatic stages due to a lack of symptoms in early disease. Systemic therapy is currently recommended as palliative treatment for patients with metastatic disease [3]. Although research has yielded advances in developing new treatment strategies, survival rates remain poor with a median overall survival (OS) of one year in advanced stages [4].

The combination of a platinum and fluoropyrimidine (5-FU) is the global standard first-line chemotherapy regimen within a non-curative setting [5]. For patients in adequate performance status (PS) a second-line systemic therapy may prolong survival and improve symptom control [6]. After platinum and 5-FU failure paclitaxel plus ramucirumab has been established as standard second-line therapy [7]. However, treatment-related neuropathy, progression during or rapid recurrence following perioperative FLOT regimen (fluorouracil, oxaliplatin, docetaxel) raise the demand for a taxane-free second-line therapy [8]. Trifluridine/tipiracil has recently been approved for patients with metastatic gastric or GEJ cancer who had received at least two previous chemotherapy regimens with a survival benefit of 2.1 months compared to placebo (median OS 5.7 months) [9].

As shown in a US-based real-world study, more than one-quarter of patients with advanced or metastatic gastric or GEJ cancer are not receiving any systemic therapy. Of the remaining three-quarters of patients who are treated, only 50% reach second-line, and less than 20% receive a third-line therapy. The latter findings clearly highlight the demand for more effective and tolerable treatment options [10].

The phase III ATTRACTION-2 trial could show improved survival outcomes for the anti-programmed death-1 (PD-1) antibody nivolumab in Asian patients with metastatic, chemotherapy-refractory gastric and GEJ cancer. Regardless of programmed death-ligand 1 (PD-L1) expression, a survival benefit of 1.2 months (median OS 5.3 months) compared to placebo has been demonstrated [11]. Efficacy and safety of nivolumab alone or in combination with ipilimumab in patients with chemotherapy-refractory gastric and GEJ cancer was investigated within the CheckMate-032 study [12].

Based on the findings from the KEYNOTE-059 trial, which showed a median OS of 5.6 months in the entire study cohort, the PD-1 inhibitor pembrolizumab received US Food and Drug Administration (FDA) approval for third-line or subsequent therapy in the subgroup harbouring a PD-L1 combined positive score (CPS) ≥ 1 [13, 14]. Furthermore, the National Comprehensive Cancer Network (NCCN) guidelines suggest pembrolizumab for second-line or subsequent therapy in patients with any microsatellite instability-high (MSI-H) or mismatch repair deficient solid tumour [3]. The percentage of MSI frequency in gastric cancer does range from 10 to 22% [15].

The Austrian Consensus on systemic therapy in patients with gastric adenocarcinoma recommends more frequent assessment of MSI and PD-L1 status, as biomarker-selected patients benefit from checkpoint inhibition in a palliative setting [16, 17].

Immune checkpoint inhibitors have been approved in advanced or metastatic gastric and GEJ cancer by the FDA as well as by the Japanese Pharmaceuticals and Medical Devices Agency based on the results of the studies listed in Table 1. Due to a lack of data in non-Asian patients, approval in this indication has not been granted by the European Medicines Agency so far. Despite pending approval, nivolumab and pembrolizumab are increasingly used off-label. A recent questionnaire survey among oncologists in China revealed that nearly 80% of prescribers used PD-1/PD-L1 inhibitors in an off-label situation. The most important criteria for off-label application were both high level evidence and indications abroad [20]. Another US-based study could show that 18% of immunotherapies were prescribed for off-label indications [21].

**Table 1:** Studies of approved PD-1 inhibitors for advanced or metastatic gastric and GEJ cancers (FDA or Japan)

Trial	ATTRACTION-2	KEYNOTE-062 Arm 1	KEYNOTE-059 Cohort 1	CT01876511 Cohort C
**PD-1 inhibitor**	Nivolumab	Pembrolizumab	Pembrolizumab	Pembrolizumab
**Treatment line**	3rd or later	1st	3rd or later	2nd or later
**Phase**	III	III	II	II
**Allocation**	Randomized, double-blind	Randomized	Single arm	Single arm
**PD-L1 status**	not assessed	positive	positive/negative	not assessed
**MS status**	not assessed	not assessed	not assessed	MSI
**Sample size**	Nivolumab: 330 (total: 493)	254	259	47
**ECOG PS**	0–1	0–1	0–1	0–1
**% Asian**	100%	27%	< 23%	4.3%
**ORR**	11.2%	15%	11.6%	47%
**Median OS (months)**	5.3	10.6	5.6	not reached
**Reference**	[11]	[18]	[14]	[19]

The aim of this study was to collect and analyse real-world data of patients with metastatic gastric and GEJ cancer treated with immune checkpoint inhibitors in a multi-institutional Western cohort.

## Materials and methods

### Study design and data collection

This is a multi-institutional retrospective chart review of clinical data in a Western population with metastatic gastric or GEJ cancer who received the PD-1 inhibitor nivolumab or pembrolizumab in a palliative setting. Nine oncologic centers in Austria participated in the collection of data. Eligible patients were aged 18 years or older; had histologically confirmed gastric or GEJ adenocarcinoma assessed by local pathology in advanced stage; and had been treated with nivolumab or pembrolizumab. The analysis was approved by the ethics committee of the provincial government of Salzburg (415-EALL/5/39–2019), the ethics committee of the Medical University of Vienna (2000/2020) and the ethics committee of the Medical University of Innsbruck (1304/2019).

### Treatment

The indication for treatment with an immune checkpoint inhibitor and type of therapy were not predefined in this retrospective analysis. Choice and scheduling of nivolumab or pembrolizumab was the sole decision of the responsible treating physician based on the findings of the ATTRACTION-2 trial, KEYNOTE-059 trial and according to NCCN guidelines [3, 11, 14]. Nivolumab was administered either at 3 mg/kg body weight or 240 mg flat dose every two weeks and pembrolizumab at 200 mg every three weeks intravenously. Palliative treatment was classified as first-line therapy with regard to evidence of metastatic disease, irrespective of the interval from perioperative chemotherapy for localized disease.

### Tumour tissue analyses

Analysis of tumour tissue was performed by the respective local pathology institute. Expression of PD-L1 was assessed by immunohistochemistry (IHC) and classified as positive if CPS was ≥1 or tumour proportion score (TPS) was ≥1%. Choice of PD-L1 scoring system was made by each center. Microsatellite status was determined by IHC, polymerase chain reaction and/or next generation sequencing.

### Statistical analyses

Baseline characteristics were analysed descriptively. PFS was calculated from the date of start of nivolumab or pembrolizumab therapy until radiologically confirmed progression or death from any cause. Patients without progression at the last contact were censored. OS was calculated from the date of start of nivolumab or pembrolizumab therapy until death from any cause. Patients alive at the last contact were censored. Overall response rate (ORR) was evaluated using Response Evaluation Criteria in Solid Tumours (RECIST 1.1) [22]. The median PFS and OS were determined using the Kaplan-Meier method. The log-rank test was used to compare survival between patient groups. The Cox proportional-hazards model was used to obtain hazard ratios and their 95% confidence intervals. Statistical analyses were performed using SPSS for Windows v23 (IBM, Armonk, NY, USA). *P*-values < 0.05 were considered to indicate statistical significance.

## Results

### Patient baseline characteristics

In total, 50 patients with metastatic gastric or GEJ cancer who had been treated with nivolumab or pembrolizumab from November 2015 until April 2020 were identified at nine oncologic centers in Austria. Baseline characteristics are listed in Table 2.

**Table 2:** Baseline characteristics at treatment initiation

Parameters	Variables	Total number	Percentages
Total number		50	
Age (years), median (range)		58 (27–87)	
	≥ 65 years	14	28%
Sex			
	male	31	62%
	female	19	38%
ECOG PS			
	0	6	12%
	1	20	40%
	≥ 2	12	24%
	unknown	12	24%
Primary tumour localisation			
	Gastric	27	54%
	Gastroesophageal junction	23	46%
Resection of primary tumour			
	yes	25	50%
	no	25	50%
Perioperative therapy			
	yes	24	48%
	no	26	52%
Number of previous palliative therapy lines			
	0	7	14%
	1	10	20%
	2	19	38%
	≥ 3	14	28%
Number of subsequent palliative therapy lines			
	0	37	74%
	1	7	14%
	≥ 2	6	12%
Time point of metastases detection			
	synchronous	25	50%
	metachronous	25	50%
Site of metastases ^1^	Peritoneum	17	34%
	Lung	11	22%
	Liver	25	50%
	Other	39	78%
Histologic subtype (Lauren classification)			
	intestinal	21	42%
	diffuse or signet ring cell	12	24%
	unspecified	17	34%
HER-2 status			
	positive	8	16%
	negative	40	80%
	unknown/missing	2	4%
Microsatellite status			
	MSI	8	16%
	MSS	31	62%
	unknown/missing	11	22%
PD-L1 expression			
	positive (CPS ≥1 or TPS ≥1%)	24	48%
	negative	13	26%
	unknown/missing	13	26%

^1^ Multiple designations possible

Median age was 58 years with a range from 27 to 87 years when immune checkpoint inhibition was initiated. One-quarter of patients was 65 years or older. The majority of our cohort (84%) had an Eastern Cooperative Oncology Group (ECOG) PS ≥ 1. Primary tumour site was well balanced between stomach and gastroesophageal junction.

Each patient had proof of metastatic disease at initiation of immunotherapy, whereby time point of metastases detection was equally distributed to synchronous (within three months of initial diagnosis) and metachronous. Among the subgroup with synchronous metastisation (*n* = 25), four patients were first treated with curative intent and developed metastases during or shortly after perioperative chemotherapy. Among the subgroup with metachronous metastisation (n = 25), most patients (*n* = 20) received perioperative therapy and underwent resection of the primary tumour. The remaining five patients received palliative treatment for localised disease (e.g., due to explicit refusal of surgery by the patient or an absolute contraindication to resection) and metastasised in the course of disease. Leading organs of secondary dissemination were peritoneum and liver.

Median number of previous palliative therapy lines was two, ranging from zero to seven. Primary treatment mostly consisted of platinum plus 5-FU in the palliative setting and FLOT in the curative setting. In two-thirds (66%) of patients, two or more palliative therapy lines prior to nivolumab or pembrolizumab had been administered. Seven patients (14%) had not been pretreated with palliative intent. About one-quarter (26%) received subsequent treatment, whereby eight patients (16%) were still on immunotherapy at last follow-up.

### Tumour characteristics

Tumour tissue was analysed by each center. All patients had histologically confirmed adenocarcinoma of the stomach or GEJ with intestinal adenocarcinoma as leading subtype. A deficient DNA mismatch repair status was detected in 16% of patients, a positive PD-L1 expression in 48%. In the nivolumab subgroup (*n* = 19), PD-L1 positivity and microsatellite instability were found in 32% (*n* = 6) and 5% (n = 1), respectively. In the pembrolizumab subgroup (*n* = 31), PD-L1 positivity and microsatellite instability were found in 58% (*n* = 18) and 23% (*n* = 7), respectively.

### Outcome

**Fig. 1:**
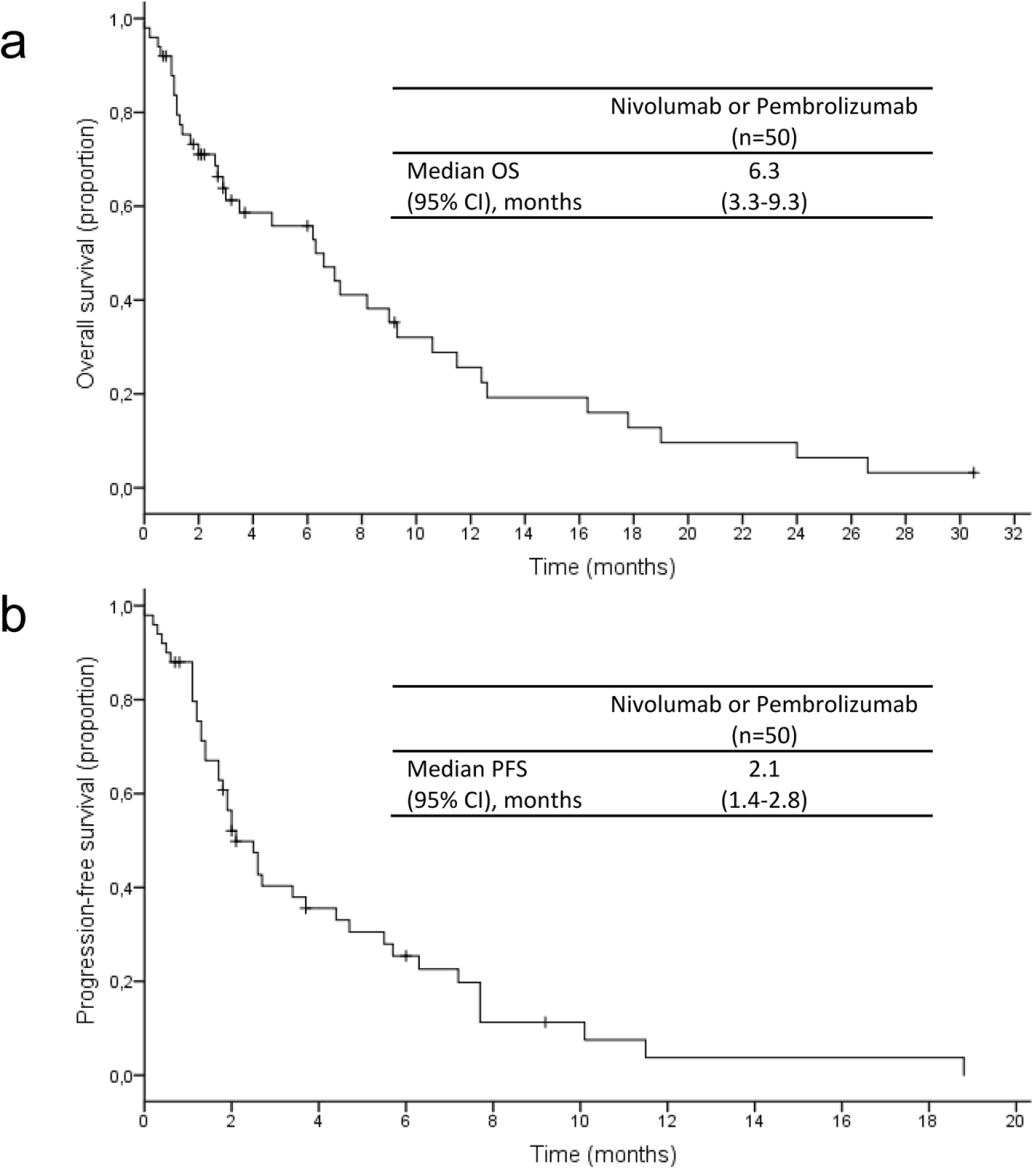
Kaplan-Meier plot of OS (a) and PFS (b). Marks on the curve indicate patients who were censored

Response evaluation could be performed in three-quarters of patients (72%, *n* = 36) at the time of data cut-off, as nine patients (18%) had deceased before first restaging and in five patients (10%), who were still on treatment, the response has not been assessed yet. Best overall responses were a partial response in five (10%) and stable disease in eleven patients (22%), resulting in a disease control rate of 32% (*n* = 16) and an ORR of 10% (*n* = 5). Only one patient discontinued immunotherapy due to treatment-related toxicity in the form of a pneumonitis grade 3 according to Common Terminology Criteria for Adverse Events (CTCAE), which occurred 1.3 months after initiation of immune checkpoint inhibition. The median PFS and OS of the entire cohort were 2.1 (95% CI: 1.4–2.8) and 6.3 (95% CI: 3.3–9.3) months, respectively (Fig. 1).

**Fig. 2:**
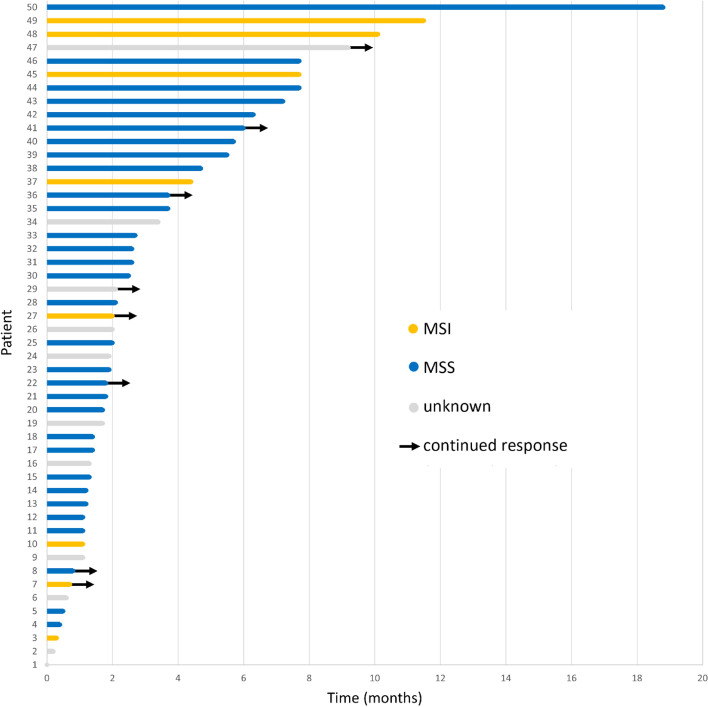
Swimmer plot of response to nivolumab or pembrolizumab according to microsatellite status

There was no statistically significant difference in median OS according to microsatellite status (MSS: 6.3 versus MSI: 11.5 months; HR = 1.21, 95% CI: 0.5–3.1; *p* = .69) or PD-L1 status (negative: 9.3 versus positive: 7.2 months; HR = 0.87, 95% CI: 0.4–2.1; *p* = .74) in patients with available data (Table 2). Median PFS was not significantly affected by microsatellite status (MSS: 2.5 versus MSI: 7.7 months; HR = 1.71, 95% CI: 0.7–4.3; *p* = .26, Fig. 2) or PD-L1 status (negative: 2.1 versus positive: 4.4 months; HR = 1.01, 95% CI: 0.5–2.3; *p* = .90).

**Fig. 3:**
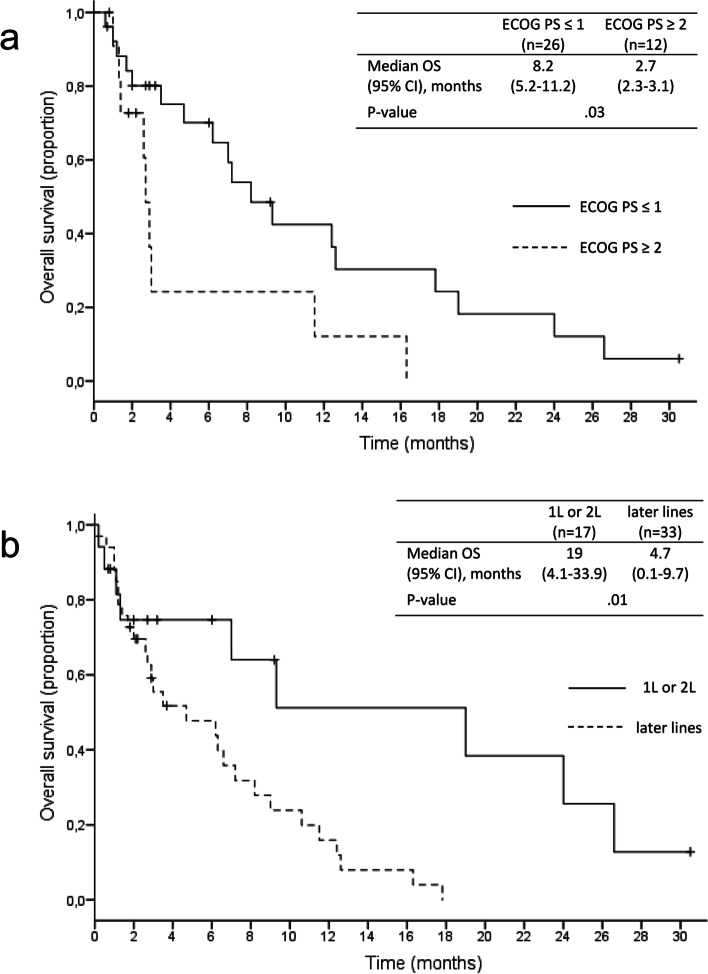
Subanalysis of OS according to ECOG PS (a) and treatment line (b). Marks on the curve indicate patients who were censored

Patients with GEJ tumours showed a significantly better outcome than those with gastric primaries (12.6 versus 6.2 months median OS; HR = 0.47, 95% CI: 0.2–0.9; *p* = .03). There was no statistically significant difference in outcome according to histological subtype (intestinal: 3.5 months versus diffuse/signet ring cell: 7.2 months versus unspecified adenocarcinomas: 16.3 months; *p* = .06). Furthermore, we found a significantly shorter median OS in patients with an ECOG PS ≥ 2 compared to those with an ECOG PS ≤ 1 (2.7 versus 8.2 months; HR = 2.50, 95% CI: 1.1–5.9; p = .03; Fig. 3a). However, there was no significant difference in PFS between the latter subgroups (1.4 versus 2.6 months; HR = 1.77, 95% CI: 0.8–3.8; *p* = .14). Also, patients who received nivolumab or pembrolizumab as first or second palliative therapy line showed a superior survival compared to later lines (19.0 versus 4.7 months; HR = 0.32, 95% CI: 0.1–0.8; *p* = .01; Fig. 3b). There was no significant difference in PFS between these subgroups (2.6 versus 2.0 months; HR = 0.67, 95% CI: 0.3–1.3; *p* = .25). Time point of metastisation (synchronous: 6.3 versus metachronous: 8.2 months; HR = 1.19, 95% CI: 0.6–2.3; *p* = .60), presence of peritoneal metastases (no: 6.2 versus yes: 7.2 months; HR = 1.24, 95% CI: 0.6–2.5; *p* = .54) and HER2 status (negative: 7.0 versus positive: 3.0 months; HR = 0.9, 95% CI: 0.4–2.2; *p* = .83) showed no significant impact on median OS.

## Discussion

In this multi-institutional retrospective analysis, we assessed 50 patients with metastatic gastric or GEJ cancer who received the PD-1 inhibitors nivolumab or pembrolizumab in a palliative setting. Nine oncologic centers in Austria took part in data acquisition.

Our cohort showed a median OS of 6.3 months (95% CI: 3.3–9.3) and PFS of 2.1 months (95% CI: 1.4–2.8), similar to the outcomes in the nivolumab arm of the ATTRACTION-2 trial (median OS and PFS: 5.3 and 1.6 months) and the pembrolizumab arm of the KEYNOTE-059 trial (median OS and PFS: 5.6 and 2.1 months) [11, 14]. The survival benefit also seems to be comparable to trifluridine/tipiracil (median OS and PFS: 5.7 and 2.0 months), which is approved as third-line therapy in metastatic gastric or GEJ cancer [9].

In contrast to existing literature, we found no statistically significant difference in median OS and PFS according to microsatellite or PD-L1 status. However, this might be due to the comparably small sample size, both unknown microsatellite and PD-L1 status in 16% of patients (*n* = 8), differing testing methods of microsatellite and PD-L1 status, as well as the heterogeneity in our cohort regarding ECOG PS, age and treatment lines.

We could observe a trend towards prolonged OS (MSS: 6.3 versus MSI: 11.5 months; HR = 1.21) and PFS (MSS: 2.5 versus MSI: 7.7 months; HR = 1.71) for the MSI subgroup, although not reaching statistical significance. This observation is consistent with well-known mechanisms of immunosurveillance. Defective mismatch repair systems lead to an excessive number of somatic mutations and presentation of neoantigens by MSI tumours, initiating infiltration by CD8 positive T-cells [23]. PD-1 inhibition can restore anti-tumour immunity after T-cell exhaustion and induce durable responses in MSI cancers [24]. In the KEYNOTE-059 study, 57.1% of the MSI subgroup experienced objective response under pembrolizumab, whereas ORR in the MSS subgroup was 9.0% [14]. Patients with advanced gastric or GEJ cancer that progressed on first-line chemotherapy were randomized to pembrolizumab monotherapy or paclitaxel in the KEYNOTE-061 study. In the paclitaxel group median OS was 8.1 months for patients with MSI tumours, while in the pembrolizumab subgroup median OS was not reached [25]. Recently, results of the phase III KEYNOTE-062 study of pembrolizumab or pembrolizumab plus chemotherapy compared to standard chemotherapy in the first-line setting were presented at the 2020 ASCO meeting. A significant survival benefit from pembrolizumab, both in combination with chemotherapy or as monotherapy, was found in patients with MSI tumours. Notably however, the ORR was higher in the subgroup receiving both chemotherapy and pembrolizumab compared to pembrolizumab alone, while OS was better with pembrolizumab alone [26]. These findings suggest that cytotoxic agents may be useful to induce a first response, while the role of prolonged administration in MSI cancer remains unclear [27]. The benefit from checkpoint inhibition in patients with advanced MSI gastric or GEJ cancer seems to be evident.

Based on the findings of the ATTRACTION-2 trial, nivolumab is recommended in Asian patients with metastatic, chemotherapy-refractory gastric and GEJ cancer regardless of PD-L1 expression. The KEYNOTE-062 study could show a survival benefit of pembrolizumab and more durable responses than chemotherapy in patients with a CPS ≥ 10, which led to consideration of pembrolizumab as first-line therapy in gastric and GEJ cancer with high CPS [26]. First results of the CheckMate-649 study were presented at ESMO meeting 2020, which demonstrated a promising survival benefit with frontline combination of chemotherapy and nivolumab in patients with PD-L1 CPS ≥ 5 [28]. Evaluation of CPS was not routinely performed in our tumour samples as choice of PD-L1 scoring system was made by each center.

One quarter of our cohort had an ECOG PS ≥ 2, which was associated with a statistically significantly shorter OS compared to patients with an ECOG PS ≤ 1 (Fig. 3a). From our own clinical experience, patients with metastatic gastric or GEJ cancer beyond palliative first-line therapy commonly present with an ECOG PS ≥ 2. This has to be put into consideration when choosing adequate treatment and avoiding potential toxicity - and could as well be a reason why less than 50% of patients with metastatic gastric or GEJ cancer receive second-line therapy [10]. Our results suggest that phase II/III studies, which exclude patients with an ECOG PS ≥ 2, might not reflect real-world clinical practice.

We found a relevant clinical benefit in patients who received nivolumab or pembrolizumab as first or second palliative therapy line compared to later lines (19.0 versus 4.7 months median OS, respectively; *p* = .01; Fig. 3b). However, these results have to be interpreted with caution as one quarter of our cohort (26%, *n* = 13) received at least one subsequent palliative therapy line. Furthermore, there was no significant difference in PFS between these subgroups (first- or second-line: 2.6 versus later line: 2 months; *p* = .25). Seven patients (14%) received immune checkpoint inhibitor therapy as first-line palliative treatment, three of which had not been pre-treated in the perioperative setting. In these cases, the choice of optimal treatment was made individually due to high PD-L1 expression, microsatellite instability, poor tolerance of cytotoxic therapy in the perioperative setting, frailty, or explicit patient wish.

Assessment of treatment toxicity according to CTCAE was not feasible by retrospective chart review. However, as only one patient discontinued treatment due to toxicity and one quarter of our cohort received at least one subsequent palliative therapy line, our data support tolerability of checkpoint inhibitors in patients with metastatic gastric or GEJ cancer.

To our knowledge, this analysis represents the largest real-world experience with checkpoint inhibitors in patients with metastatic gastric or GEJ cancer in a Western cohort outside a clinical trial and our results confirm feasibility of treatment with nivolumab or pembrolizumab in this population. As patient profiles in clinical practice may substantially differ from those in randomized clinical trials, we believe that our data are a meaningful contribution to current knowledge.

## Conclusions

In this multi-institutional retrospective analysis of checkpoint inhibitors in advanced gastric/GEJ cancer in a real-world Western cohort, we could show a similar survival benefit compared to larger phase II/III trials with differing patient characteristics. Contrary to existing literature, there was no statistically significant difference in median OS according to microsatellite or PD-L1 status. However, a trend towards prolonged PFS and OS in the microsatellite instability high subgroup could be observed. Nevertheless, our study is limited by the small number of patients and its retrospective character. We are looking forward to further phase III trials investigating checkpoint inhibitors in Western patients with metastatic gastric and GEJ cancer to confirm clinical efficacy.

## Data Availability

The datasets used and/or analysed during the current study are available from the corresponding author on reasonable request.

## Abbreviations

GEJ: Gastroesophageal junction
PFS: Progression-free survival
OS: Overall survival
PS: Performance Status
5-FU: Fluoropyrimidine
PD-1: Programmed death-1
PD-L1: Programmed death-ligand 1
FDA: Food and Drug Administration
CPS: Combined positive score
NCCN: National Comprehensive Cancer Network
MSI-H: Microsatellite instability-high
IHC: Immunohistochemistry
TPS: Tumour proportion score
RECIST: Response Evaluation Criteria in Solid Tumours
ECOG: Eastern Cooperative Oncology Group
ORR: Overall response rate
CTCAE: Common Terminology Criteria for Adverse Events
